# Improvement of HAART in Brazil, 1998–2008: a nationwide assessment of survival times after AIDS diagnosis among men who have sex with men

**DOI:** 10.1186/s12889-015-1530-y

**Published:** 2015-03-07

**Authors:** Monica Malta, Cosme M F P da Silva, Monica MF Magnanini, Andrea L Wirtz, André R S Perissé, Chris Beyrer, Steffanie A Strathdee, Francisco I Bastos

**Affiliations:** Department of Epidemiology, Johns Hopkins Bloomberg School of Public Health, 615 N. Wolfe Street E7152, Baltimore, MD 21205 USA; Oswaldo Cruz Foundation - FIOCRUZ, Sergio Arouca National School of Public Health, Department of Social Sciences, Rua Leopoldo Bulhoes, 1480 suite 905 Manguinhos, Rio de Janeiro, RJ 21041-210 Brazil; Department of Epidemiology and Quantitative Methods in Health, Oswaldo Cruz Foundation - FIOCRUZ, Sergio Arouca National School of Public Health, Rua Leopoldo Bulhoes, 1480- suite 802 Manguinhos, Rio de Janeiro, 21041-210 Brazil; Federal University of Rio de Janeiro, Institute of Public Health Studies, Praça da Prefeitura Universitária Cidade Universitária, Rio de Janeiro, 21941-598 Brazil; Department of Emergency Medicine, Johns Hopkins Bloomberg School of Public Health Department of Epidemiology, Johns Hopkins Medical Institute, 615 N. Wolfe St, E 7143, Baltimore, MD 21205 USA; Department of Biological Sciences, Rua Leopoldo Bulhoes, Oswaldo Cruz Foundation- FIOCRUZ, National School of Public Health, 1480, suite 21 Manguinhos, Rio de Janeiro, RJ 21041-210 Brazil; School of Medicine, University of California, UCSD 9500 Gilman Drive La Jolla, San Diego, CA 92093-0507 USA; Oswaldo Cruz Foundation - FIOCRUZ, Biblioteca de Manguinhos suite 229 Av. Brasil 4365, Rio de Janeiro, 21045-900 Brazil

**Keywords:** HIV, AIDS, HAART (Highly active antiretroviral therapy), Men who have sex with men (MSM), Survival analysis, Brazil

## Abstract

**Background:**

In 1996, Brazil became the first developing country to provide free, universal access to HAART, laboratory monitoring, and clinical care to any eligible patient. As of June 2014, approximately 400,000 patients were under treatment, making it the most comprehensive HIV treatment initiative implemented thus far in a middle-income country, worldwide. The Brazilian epidemic is highly concentrated among men who have sex with men (MSM).

**Methods:**

Four national information systems were combined and Cox regression was used to conduct retrospective cohort analysis of HAART availability/access on all-cause mortality among MSM diagnosed with AIDS reported to the information systems between 1998–2008, adjusting for demographic, clinical, and behavioral factors and controlling for spatially-correlated survival data by including a frailty effect. Multiple imputation by chained equations was used to handle missing data.

**Results:**

Among 50,683 patients, 10,326 died during the 10 year of period. All-cause mortality rates declined following introduction of HAART, and were higher among non-white patients and those starting HAART with higher viral load and lower CD4 counts. In multivariable analysis adjusted for race, age at AIDS diagnosis, and baseline CD4 cell count, MSM diagnosed in latter periods had almost a 50% reduction in the risk of death, compared to those diagnosed between 1998–2001 (2002–2005 adjHR: 0.54, 95% CI:0.51-0.57; 2006–2008 adjHR: 0.51, 95% CI:0.48-0.55). After controlling for spatially correlated survival data, mortality remained higher among those diagnosed in the earliest diagnostic cohort and lower among non-white patients and those starting HAART with higher viral load and lower CD4 lymphocyte counts.

**Conclusions:**

Universal and free access to HAART has helped achieve impressive declines in AIDS mortality in Brazil. However, after a 10-years follow-up, differential AIDS-related mortality continue to exist. Efforts are needed to identify and eliminate these health disparities, therefore improving the Brazilian response towards HIV/AIDS epidemic.

## Background

By the end of 2013, 35 million [33.2 million-37.2 million] people were living with HIV worldwide and 1.5 million [1.4 million-1.7 million] AIDS-related deaths were reported in the latest Epidemiological Bulletin released by UNAIDS. The introduction of combination antiretroviral therapies has led to dramatic improvements in AIDS-related morbidity and mortality in countries where this treatment is available [1].

Highly active antiretroviral therapy (HAART) significantly improves the prognosis of HIV-infected persons by reducing HIV viral load, increasing CD4+ lymphocyte counts and delaying progression to AIDS, ultimately reducing mortality rates [2,3].

Pre-exposure prophylaxis (PrEP) for HIV infection has greatly improved the field of HIV prevention [4], and a recent systematic review suggests its efficacy among high-risk groups such as men who have sex with men – MSM [5].

Early detection and management of HIV infection are key challenges worldwide, but a growing body of evidence supports the immediate use of HAART to maintain CD4 count and functionality, limit the size of the HIV reservoir, and reduce the risk of onward viral transmission [6]. Following such groundbreaking results, Brazil has recently announced plans to adopt the “Treatment as Prevention” strategy to curb HIV and AIDS [7].

However, AIDS-related deaths continue to occur, even in high-income countries, and the emergence of drug-resistant HIV variants and drug toxicities remain major barriers and challenges to successful long-term antiretroviral efficacy [8,9].

Although stable, on average, the epidemic in Central and South America is currently concentrated in specific groups, mainly among MSM [1,10]. In Brazil MSM accounted for more than 32% of the cumulative reported cases of AIDS from 1980 to 2010 among men older than 13 years of age [11]. Data from a Brazilian meta-analysis indicated a pooled HIV prevalence of 13.6% (95% CI:8.2-20.2) among MSM [12]. National data from sexually transmitted infections’ clinics in Brazil demonstrated a 1.7% overall prevalence for HIV among clinic attendees [11]. However, when stratified for subgroups defined by sexual exposure, prevalence among MSM was found to be 5.7%, compared to 1.1% among heterosexual men. Data from three voluntary counseling and testing services reported 24.8% prevalence among MSM and 4.3% among heterosexual men [13]. HIV incidence at VCT units in Rio de Janeiro, Brazil, indicated incidence up to 11 times higher among MSM when compared to heterosexual men [13,14].

Brazil was the first middle-income country to provide full access to HAART, laboratory monitoring and clinical care at no cost at the point of health care delivery to any eligible patient, since 1996 [15]. As of June 2014, approximately 400,000 patients were receiving HAART in Brazil [11], making it the most comprehensive HIV treatment initiative implemented thus far in a middle-income country, worldwide [16].

Recent Brazilian observational studies have brought essential information about mortality patterns among HIV-positive patients under follow-up in Brazil’s referral centers [17,18]. However, clinical cohorts from selected referral centers do not assess real-life conditions found in busy, understaffed public health clinics scattered all over Brazil, a country where social and regional heterogeneities are very relevant in the field of AIDS care [19,20], as well as health care in a broad sense [21].

Herein, we report differences in survival from AIDS diagnosis according to period of AIDS diagnosis, within the unique Brazilian setting. The study evaluated all MSM receiving treatment in the country between 1998 and 2008, therefore avoiding selection bias present in analyses from specific subsets (such as referral centers from major southeastern metropolitan areas, particularly Rio de Janeiro and Sao Paulo) of the country’s population living with HIV/AIDS.

## Methods

This study utilized four databases comprising different longitudinal information of all people living with HIV/AIDS (PLWHA) under treatment and care through the Brazilian public health system. These databases comprise all patients receiving any antiretroviral medicine in Brazil since such medicines are not procured, purchased, or delivered by private health facilities or pharmacies for patients who are not hospitalized.

These databases contain the core information of Brazil’s surveillance system. Their basic characteristics are briefly summarized as follows:

i. Socio-demographic and basic clinical (for the sake of diagnosis) information on AIDS cases (SINAN-AIDS - Information System for Notifiable Diseases/AIDS);
ii. Data from exams conducted in the network of accredited public laboratories, particularly TCD4+/TCD8 lymphocyte counting and HIV viral load (SISCEL - National Database for Laboratory Tests);
iii. Information about monthly ARV refills and therapeutic regimen changes over time (SICLOM - Logistics Control System of ARV Medicines); and
iv. The date and cause/s of death (SIM - National Mortality Database).

Whereas patients may seek private medical care and perform laboratory exams in private facilities, all PLWHA receive their antiretroviral drugs through SUS (Brazil’s Unified Public Health System), the single accredited source of ARVs in Brazil, and should be included in SICLOM database. At the time we conducted our analysis, SICLOM data have been updated to improve the comprehensiveness of collected data, which tend to be limited to the core information which is essential for medicines dispensing at the several health services belonging to the network of accredited public health units. Thus, to utilize data from all regions, we could not include additional information such as measures of adherence, side effects, drug regimen shifts, etc. as these data were not yet available for all regions and services.

Data from the entire population of PLWHA with AIDS diagnosis between 1998 and 2008, whose transmission category was homo/bisexual contact were extracted from those databanks and merged. The databank linkage procedures have been described in detail elsewhere [15].

For this analysis, participants were included if they were diagnosed as having AIDS between January 1, 1998 and December 31, 2008, and if their most probable route of HIV infection was male-male unprotected anal intercourse. Those patients were included because we aimed to analyze differences in survival from AIDS diagnosis among the MSM population, for which higher rates of initiation and better adherence to HAART during three different AIDS diagnosis periods: 1998–2001, 2002–2005, and 2006–2008 have been documented in the literature and by our own exploratory analyses.

The final merged database comprised the following covariates: date of birth, race, date of AIDS diagnosis, dates of initiation of HAART, CD4 counts and viral load at diagnosis, cause(s) and date of death. CD4 counts at initial diagnosis were classified into three categories: < 200 cells/mm^3^, 200–350 cells/mm^3^, and >350 cells/mm^3^. Viral load at initial diagnosis was classified as <4.00 log_10_ copies, 4.00-5.00 log_10_ copies, and >5.00 log_10_ copies.

### Statistical analyses

The primary endpoint of interest in the present study was all-cause mortality during 1998–2008. Survival was calculated as the time elapsed from date of AIDS diagnosis until the date of death. Individuals who remained alive were censored at the end of data collection period (December 31, 2008). Time from AIDS diagnosis was transformed from days to years since diagnosis. Since analyses were based on secondary data, we did not have access to information on clinical visits; therefore we were not able to calculate losses to follow-up.

Kaplan-Meier curves were fitted to compare probabilities of survival according to selected period of diagnosis and CD4 count at diagnosis using the log-rank test. Cox proportional hazard regression modeling was used to examine covariates associated with survival among the following: age at AIDS diagnosis, race, period of AIDS diagnosis (1998–2001; 2002–2005; 2006–2008), first CD4+ lymphocyte count, HAART uptake, and first HIV-RNA viral load exam. The likelihood ratio test was used to select variables to be entered in the Cox models, adjusting for potential confounders. The assumption of proportional hazards was evaluated through the examination of Schoenfeld residuals [22].

Hazard rates are strongly influenced by selection effects operating in the population (e.g. higher availability of referral centers in a given locality), and individuals surviving up to a certain time will on average be less frail than the original population, therefore unobserved individual heterogeneity (or frailty) was also taken into account [23].

With 8.5 million square kilometers and over 202 million inhabitants according to the National Census, 2010, updated as of July 2013 [24] and regular updates issued by the Brazilian Institute for Geography and Statistics (IBGE), Brazil is the largest and most populous country in Latin America, and faces deep health and socio-economic differences between and within each region. In an attempt to account for the heterogeneity between and within different Brazilian regions and localities, Cox regression analyses were repeated with the introduction of a random effect term to assess the potential association between survival times within a cluster – state or municipality of residency – using gamma-distributed frailty [23]. A frailty indicator >1 denotes a faster event (death) rate, while groups will frailty <1 survival tend to be longer [25].

To estimate confidence intervals for Cox's proportional hazards models, robust methods were used. Maximum penalized likelihood estimation was used to fit frailty models. To handle missing data, we implemented multiple imputation by chained equations using classification and regression trees (MICE-CART) as the conditional models for imputation [26]. This method can result in more plausible imputations, and hence more reliable inferences, in complex settings than the naive application of standard sequential regression imputation techniques.

Creating multiple imputations, as opposed to single imputations, accounts for the statistical uncertainty in the imputations. In addition, the chained equations approach is very flexible and can handle variables of varying types (e.g., continuous or binary), and has been reported as very reliable when used in different datasets [27,28]. All analyses were performed using R software version 3.0.2 (library survival) and Stata 13.0 (College Station, TX) [29].

#### Ethical approval

No identifiable personal data were used for this study. The dataset used in the study is not openly available. Permission to use non-identifiable, individual data extracted from HIV/AIDS administrative databases was granted by the Brazilian Ministry of Health, Department of STDs, AIDS and Viral Hepatitis. Ethical approval for use of encrypted and aggregated data was also obtained from the Oswaldo Cruz Foundation Research Ethics Committee (Protocol CEP/ENSP 179/08) and the Brazilian National Research Ethics Committee (CONEP 15308).

## Results

### Characteristics of the patients

Between January 1 1998 and December 31 2008, 50,683 MSM were diagnosed with AIDS in Brazil. Of these, 20,702 (40.9%) were diagnosed between 1998–2001, 19,280 (38.0%) in 2002–2005, and 10,701 (21.1%) in the last period under analysis, 2006–08. The overall mean age at AIDS diagnosis was 35.4 years (SD: 9.5). Data on race from 39.0% of our sample was missing. Although all patients had a confirmed AIDS (clinical) diagnosis, there were no information respecting 29.5% of the CD4 counts and 42.5% HIV-1 RNA viral load determinations in the merged database (Table 1). Due to the significant amount of missing data, MICE-CART multiple imputation was conducted before running survival analysis.

**Table 1:** **Sociodemographic and clinical characteristics of the study patients**

		**Diagnostic period**		
	**Total**	**1998** − **2001**	**2002** − **2005**	**2006** − **2008**
Subjects (N, %)	50,683	20,702	19,280	10,701
Follow-up time (person-years)	249,656.30	149,494.24	84,776.07	15,385.97
Age at diagnosis (years)				
Mean ± SD	35.4 ± 9.5	35.3 ± 9.2	35.3 ± 9.5	35.8 ± 9.9
Range	18 – 89	18 – 89	18 – 86	18 – 85
Ethnicity				
Caucasian	18,399 (36.3)	3,087 (14.9)	9,454 (49.0)	5,858 (54.8)
Mulatto (mixed white and black)	9,290 (18.3)	1,408 (6.8)	4,701 (24.4)	3,181 (29.7)
Black	2,912 (5.8)	478 (2.3)	1,520 (7.9)	914 (8.5)
Others (Asian, Indigenous…)	307 (0.6)	64 (0.3)	132 (0.7)	111 (1.0)
Unspecified	19,775 (39.0)	15,666 (75.7)	3,473 (18.0)	637 (6.0)
Deaths^a^	10.326	5.791	3.258	1.277
Have at least one CD4 exam available on SISCEL				
No	14,934 (29.5)	8.081 (39.0)	4.082 (21.2)	2,771 (25.9)
Yes	35,749 (70.5)	12.621 (61.0)	15.198 (78.8)	7,930 (74,1)
CD4 lymphocytes, cells/mm^3^ (first exam available)^b^				
Mean ± SD	298.1 ± 185.3	324.8 ± 179.3	289.5 ± 191.7	261.9 ± 177.0
Range	0 – 999	0 – 998	0 – 998	0 – 999
Have at least one HIV-1 RNA Viral load exam available on SISCEL				
No	21,562 (42.5)	11,097 (53.6)	6,601 (34.2)	3,864 (36.1)
Yes	29,121 (57.5)	9.605 (46.4)	12,679 (65.8)	6,837 (63.9)
HIV RNA in plasma, log_10_ copies/ml (first exam available)^c^				
Mean ± SD	4.46 ± 1.01	4.38 ± 1.04	4.46 ± 1.02	4.60 ± 0.91
Range	1.70 – 6.69	1.70 – 6.67	1.70 – 6.69	1.70 – 6.69

^a^All causes of death; ^b^Among those with at least one CD4 exam; ^c^Among those with at least one viral load exam; SISCEL: National Database for Laboratory.

There were 10,326 deaths during the 10 year period. The median follow-up times for patients in three diagnosis periods are: period 1998–2001: 8.36 years; period 2002–2005: 4.68 years; and period 2006–2008: 1.44 years.

**Figure 1:**
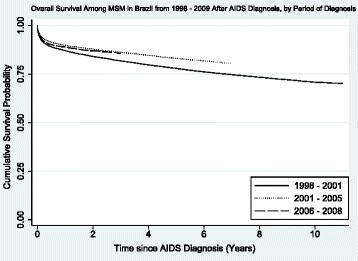
**Overall Survival Among MSM in Brazil from 1998**–**2009 After AIDS Diagnosis**, **by Period of Diagnosis.**

In Kaplan-Meier analyses stratified by diagnostic period, those diagnosed in the first period had significantly lower survival since AIDS diagnosis (p < 0.0001), with visible improvements among patients from the second and third cohort period (p < 0.0001). Taking into consideration the short follow-up time for participants in diagnostic period 2006–2008, there appeared to be little difference in survival between the last two diagnostic periods (Figure 1).

In the unadjusted analysis, the risk of death was lower for both periods 2002–2005 (HR: 0.74; 95% CI:0.71-0.77; p < 0.001) and 2006–2008 (HR: 0.81; 95% CI:0.76-0.86; p < 0.001), compared to the first period of diagnosis (1998–2001). The unadjusted HR for the period 2006–2008 was higher (HR: 1.09; 95% CI:1.02-1.17; p < 0.007) when compared to the reference of 2002–2005 to assess difference between latter periods (data not shown).

### Predictors of survival

Controlling for other variables, the risk of death among latter cohorts of diagnosis was lower when compared to the cohort diagnosed between 1998–2001 (Period 2002–2005 adjHR: 0.54, 95CI:0.51-0.57; Period 2006–2008 adjHR: 0.51, 95%CI:0.48-0.55). There was no difference between the cohort diagnosed in 2006–2008 compared to the cohort diagnosed in 2002–2005, after adjustment in the Cox model (adjHR: 0.95; 95% CI:0.89-1.02). Additional predictors of longer survival included younger age at diagnosis and higher CD4 counts at diagnosis. Patients with an HIV-RNA viral load >5log_10_ copies/ml at diagnosis had a higher risk of death, as well as non-White patients (Table 2).

**Table 2:** **Hazard ratios of mortality according to baseline variables among MSM patients diagnosed with AIDS in Brazil**, **1998**–**2008**

	**Adjusted**		**Adjusted by state frailty effect**	
**Predictor**	**HR**	**95% CI**	**HR**	**95% CI**
Age (per 10 year increase)	1.13*	(1.11 - 1.16)	1. 13*	(1.11 - 1.16)
Race**				
White	Reference			Reference
Non-white	1.15*	(1.09 - 1.21)	1.29*	(1.22 - 1.37)
Unspecified	0.89*	(0.84 - 0.94)	1.02	(0.96 - 1.08)
Period of diagnosis^t^				
1998-2001	Reference			Reference
2002-2005	0.54*	(0.51 - 0.57)	0.55*	(0.52 - 0.58)
2006-2008	0.51*	(0.48 - 0.55)	0.52*	(0.49 - 0.56)
CD4 cell count at diagnosis (cells/mm^3^)				
< 200	Reference			Reference
200 - 350	0.65*	(0.63 - 0.68)	0.67*	(0.65 - 0.70)
>350	0.20*	(0.18 - 0.21)	0.19*	(0.17 - 0.20)
HIV-1 RNA (log_10_ copies)				
<4.00	Reference			Reference
4.00-5.00	1.28*	(1.19 - 1.37)	1.22*	(1.14 - 1.31)
>5.00	4.68*	(4.40 - 4.98)	5.26*	(4.94 - 5.61)
Estimated frailty variance		---		0.099*

Hazard Ratio (95% Confidence Interval); *p-value < 0.001; Cox proportional hazards model adjusted for all variables listed in the table. ^t^Comparing period of diagnosis in 2006–2008 to the reference of 2002–2005: adjusted HR: 0.95 (95% CI: 0.89 - 1.02; p = 0.144); model adjusted for state frailty effect: HR: 0.94 (95% CI: 0.88 - 0.01; p = 0.085).

Upon multivariate Cox frailty regression that accounted for a random effect associated with the Brazilian state where patients lived (Table 2), the increased risk of death observed in patients diagnosed in the first period of time persisted. Both higher CD4 cells counts and lower HIV-1 RNA viral load remained as significant predictors of survival. Non-White patients remained with a higher risk of death; however, there was no longer a difference between white participants and those with unspecified race.

The estimated frailty variance was significantly different from zero, suggesting that the risk of death after AIDS diagnosis was heterogeneous among subjects living in different states (Table 2). We adjusted the final model with and without MICE-CART imputation procedure; however there were no relevant influence in the results (data not shown).

**Figure 2:**
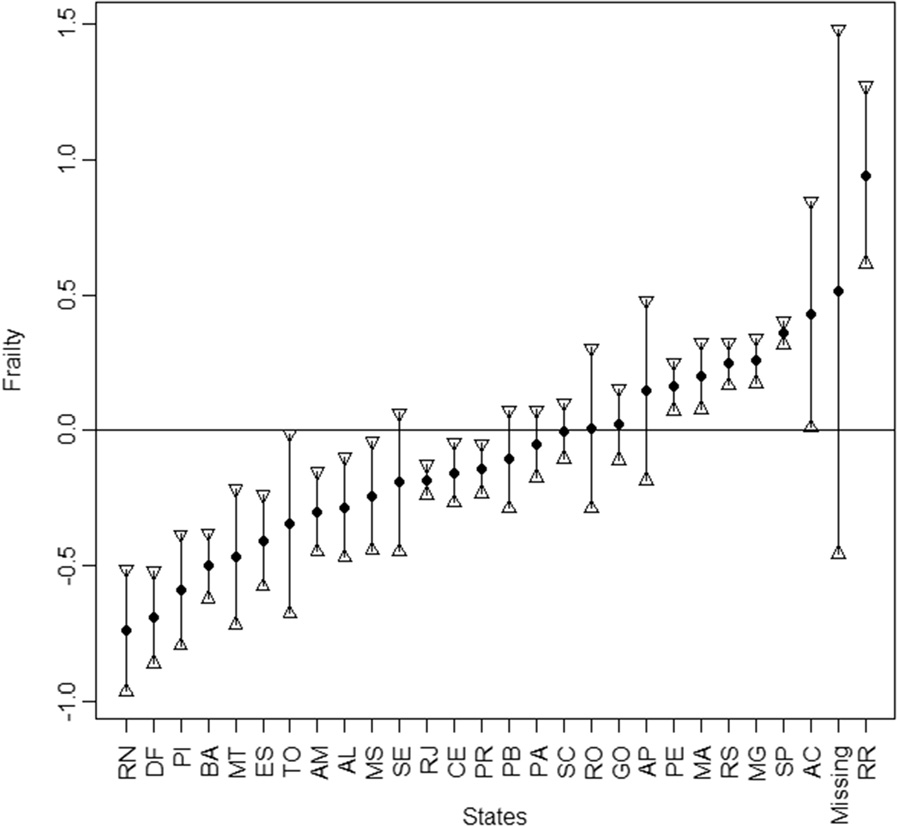
**Frailty estimates of relative risks of state showing the point estimate** (**circle**) **and a confidence interval of 95%.** Both the interval and the distribution are skewed because the relative risks in an exponential function of the gamma estimate. Abbreviations: Acre (AC); Alagoas (AL); Amapá (AP); Amazonas (AM); Bahia (BA); Ceará (CE); Distrito Federal (DF); Espírito Santo (ES); Goiás (GO); Maranhão (MA); Mato Grosso (MT); Mato Grosso do Sul (MS); Minas Gerais (MG); Pará (PA); Paraíba (PB); Paraná (PR); Pernambuco (PE); Piauí (PI); Rio de Janeiro (RJ) ; Rio Grande do Norte (RN); Rio Grande do Sul (RS); Rondônia (RO); Roraima (RR); Santa Catarina (SC); São Paulo (SP); Sergipe (SE); Tocantins (TO).

Greater frailty effects (and therefore greater mortality) were found in larger states located in the Tropical Rain Forest region and in states located in the Northeast, Brazil’s poorest region. On the other hand, industrialized areas from the Southern and Southeastern regions accounted for the smallest frailty effect (Figure 2).

## Discussion

This is the first Brazilian nationwide analysis of survival after AIDS diagnosis among MSM. Our study compared different time periods after the full introduction of HAART over the whole country, following the approval of the federal legislation regulating the right to the universal access to antiretroviral medicines for people living with AIDS, as of the end of 1996 [29]. Improvements in survival were observed for latter periods of diagnosis, with almost 50% reduction in risk of death when compared to the earliest cohort. Protective effects were also observed with lower age and higher CD4 classifications at initial diagnosis.

Previous papers documented the dramatic improvement of survival before and after HAART [30], and identified lower mortality rates among PLWHA from Brazil, when compared with patients from other Latin American countries [31]. Changes in the main causes of death from AIDS to non-AIDS related conditions have also been identified [18,32], however, those studies were conducted in a pool of referral centers or in a single referral center, and none of them assessed nationwide data, taking in consideration their pronounced heterogeneity.

The observed improvements in the latter periods of diagnosis compared to those diagnosed between 1998–2001 are favorable. No difference, however, has yet been detected between those diagnosed in 2006–2008 compared to those diagnosed in 2002–2005. This finding may be attributed to barriers and problems in the current treatment program and the concrete operations of the respective network of public health services. Among the most relevant of such challenges, some are highlighted as follows: i) the increasing pressures over costs [33], challenging the very sustainability of universal access over time; ii) the deep heterogeneity of the Brazilian network of health services, either in the specific field of AIDS management and care [20] or from the perspective of the health system in a broad sense [21]; and, iii) last but not the least, the late presentation of a substantial fraction of patients [19]. Nonetheless, it is important to note that the median follow-up time for patients diagnosed in 2006–2008 is substantially shorter and future research may observe improvements after longer durations of follow-up of this cohort.

Other problems to be tackled by clinicians and health managers include the progressive, albeit moderate, increase of secondary resistance [34] and the permanent challenge posed by side effects of different antiretroviral medications, such as lipodystrophy and metabolic syndromes [35]. However, despite all such caveats and difficulties, the present paper documents a progressive increase of survival times among gay men over successive periods of the decade 1998–2008. Similar findings were identified by a multi-site study comprising data from several North American cohorts. According to the study conducted by Samji and colleagues [29], who analyzed data from almost 23,000 treatment-naive ART patients from the MSM population, the life-expectancy at age 20 years increased with calendar time, from 53.3 in 2000–2002 to 69.3 in 2006–2007. Future Brazilian studies should include more recent timeframes, as well as additional information about HAART uptake and treatment adherence.

## Conclusions

Our statistical modeling incorporated frailty effects and demonstrated a pronounced effect of geographic, social and/or health care quality upon survival times. It is not possible to disentangle the independent effects of each one of these factors; however, our study highlighted the negative impact on survival among patients living in less industrialized and most deprived regions of Brazil. Patients living in such areas are likely to suffer from an adverse and synergic effect of poverty and lack of social support, receiving less than optimal care in settings where health services are scarce, overburdened, less accessible and of lower quality [21].

As in any analysis of large secondary datasets, there is a trade-off between the augmented comprehensiveness of patient data under analysis and the deficiencies in terms of coverage and quality of databases. In this sense, the missing information identified in the current study constitutes serious limitations, not only in terms of the resulting analyses, but also from the broader perspective of monitoring and policymaking.

Such barriers and caveats must be progressively overcome by policymakers, managers, health professionals and the civil society in a continental-sized, deeply heterogeneous country, housing the most comprehensive network of ARV dispensing units worldwide. With increasing costs and the pressing need to switch therapeutic regimens in order to minimize both HIV resistance and different adverse effects of ARVs, substantial improvement of information systems is mandatory and urgently needed, as already highlighted by Grangeiro and colleagues [36].

The improvement in survival from the first period of HAART is undeniable and should be commended. However, results should be viewed in light of some study limitations. First, the process of merging four national databases resulted in missing data among key variables, such as CD4+ lymphocyte counts and viral load measures. Such limitations forced us to conduct multiple imputation (MICE-CART) of several variables before running the survival analysis. We did not conduct MICE-CART for race, a key variable with a significant proportion of missing information. This was a methodological and operational decision: patients with private health insurance usually have scarce information available on national datasets and our MICE-CART could not be accurately conducted with the available information. This problem calls for an urgent need to provide better training of professionals and clerks in charge of monitoring the Brazilian AIDS epidemic and to increase the accountability and consistency of such systems. A second limitation lies in the comparison of survival across time periods of initial diagnosis. Because our dataset is limited to 1998 to 2008, length of follow-up is shorter for those diagnosed in 2002–2005 and 2006–2008, limiting comparisons in survival after six to ten years following diagnosis. Nonetheless, these data are sufficient to allow for comparisons of trends in survival across different periods of diagnosis and CD4 count to AIDS diagnosis during the early years of HAART.

Brazil has been a worldwide leader in the global efforts to curb AIDS and to provide a standard of care and treatment for PLWHA at no cost at the point of delivery. Notwithstanding, the concerted effort from across different sectors of government, community leadership, and the civil society at large may be seriously compromised in the absence of a comprehensive and accurate monitoring system. Although our analyses documented some progress since the early years of HAART, current levels of missing information remain unacceptably high - a critical challenge as we enter in the third decade of the HIV/AIDS epidemic, with more complex and costly treatments, more patients under follow-up and more data being collected.
